# TLR-4 cooperates with Dectin-1 and mannose receptor to expand Th17 and Tc17 cells induced by *Paracoccidioides brasiliensis* stimulated dendritic cells

**DOI:** 10.3389/fmicb.2015.00261

**Published:** 2015-03-31

**Authors:** Flávio V. Loures, Eliseu F. Araújo, Claudia Feriotti, Silvia B. Bazan, Vera L. G. Calich

**Affiliations:** Departamento de Imunologia, Instituto de Ciências Biomédicas, Universidade de São Paulo, São Paulo, Brazil

**Keywords:** *Paracoccidioides brasiliensis*, innate immunity, toll-like receptors, mannose receptor, Dectin-1 receptor, Th17, Tc17

## Abstract

The concomitant use of diverse pattern recognition receptors (PRRs) by innate immune cells can result in synergistic or inhibitory activities that profoundly influence anti-microbial immunity. Dectin-1 and the mannose receptor (MR) are C-type lectin receptors (CLRs) previously reported to cooperate with toll-like receptors (TLRs) signaling in the initial inflammatory response and in the induction of adaptive Th17 and Tc17 immunity mediated by CD4^+^ and CD8^+^ T cells, respectively. The protective immunity against paracoccidioidomycosis, the most prevalent fungal infection of Latin America, was previously shown to be influenced by these T cell subsets motivating us to study the contribution of TLRs, Dectin-1, and MR to the development of Th17/Tc17 immunity. First, curdlan a specific Dectin-1 agonist was used to characterize the influence of this receptor in the proliferative response and Th17/Tc17 differentiation of naïve lymphocytes induced by *Paracoccidioides brasiliensis* activated dendritic cells (DCs) from C57BL/6 mice. Then, wild type (WT), Dectin-1^–/–^, TLR-2^–/–^, and TLR-4^–/–^ DCs treated or untreated with anti-Dectin-1 and anti-MR antibodies were used to investigate the contribution of these receptors in lymphocyte activation and differentiation. We verified that curdlan induces an enhanced lymphocyte proliferation and development of IL-17 producing CD4^+^ and CD8^+^ T cells. In addition, treatment of WT, TLR-2^–/–^, and TLR-4^–/–^ DCs by anti-Dectin-1 antibodies or antigen presentation by Dectin-1^–/–^ DCs led to decreased lymphoproliferation and impaired Th17 and Tc17 expansion. These responses were also inhibited by anti-MR treatment of DCs, but a synergistic action on Th17/Tc17 differentiation was mediated by TLR-4 and MR. Taken together, our results indicate that diverse TLRs and CLRs are involved in the induction of lymphocyte proliferation and Th17/Tc17 differentiation mediated by *P. brasiliensis* activated DCs, but a synergist action was restricted to Dectin-1, TLR-4, and MR.

## Introduction

Th17 cells, a recently identified subset of CD4^+^ T cells, are involved in the production of inflammatory cytokines, including IL-17 (IL-17A and IL-17F) and IL-22. They mediate the immune response against several pathogens but are also potent inducers of tissue inflammation and pathogenesis of autoimmune diseases (Liang et al., 2006; Wilson et al., 2007). TGF-β and IL-6 are the main inducers of Th17 differentiation in mice, and STAT3, RORγt, and RORα are the transcription factors involved in this process. The cytokines produced by Th17 cells stimulate a variety of cells, including polymorphonuclear neutrophils, which can participate both in pathogen clearance and in the induction of tissue pathology caused by excessive cell activation (Korn et al., 2009). IL-22 is particularly involved in the activation and repair of epithelial barriers (De Luca et al., 2010). IL-17-producing cells are crucial for antifungal defense, and fungi are particularly strong inducers of Th17 responses (Levitz, 2009; Cypowyj et al., 2012; Drewniak et al., 2013; Murdock et al., 2014). During fungal infections, Th17 cells promote fungal clearance (Pandiyan et al., 2011; Hernández-Santos and Gaffen, 2012; Hernández-Santos et al., 2012; Perez-Nazario et al., 2013) but have also been associated with excessive inflammation and tissue pathology (Zelante et al., 2007). Recently, it was demonstrated that IL-17 regulates systemic immunity by controlling the functional competence of NK cells (Bär et al., 2014) and promoting vaccine-induced host protection against fungi and other pathogens (Lin et al., 2009; Sellge et al., 2010; Wüthrich et al., 2011).

Several reports have shown the importance of Dectin-1, a C-type lectin receptor (CLR) that recognizes β-1-3-glucans present on the cell wall of many medically important fungi, in the host immune response against fungal pathogens (Hohl et al., 2005; Taylor et al., 2007). Dectin-1 signaling has been associated with pro-inflammatory innate responses and in the expansion of Th17 immunity against these pathogens (Cheng et al., 2011; Carvalho et al., 2012). Activation of Dectin-1 by specific agonists stimulates the maturation of dendritic cells (DCs) and subsequent production of IL-6, TNF-α, and IL-23, which participate in the polarization of naïve CD4^+^ T cells into effector CD4^+^IL-17^+^ cells (Acosta-Rodriguez et al., 2007; LeibundGut-Landmann et al., 2007). Interestingly, the activation of DCs with specific agonists of Dectin-1 may also determine the conversion of regulatory T (Treg) cells, which are mainly induced by TGF-β signaling, into IL-17-producing T cells (Osorio et al., 2008).

Paracoccidioidomycosis (PCM) is a systemic granulomatous disease caused by the dimorphic fungus *Paracoccidioides brasiliensis* and constitutes the most relevant deep mycosis in Latin America. In humans and murine models of PCM, resistance to disease is associated with the secretion of IFN-γ and other Th1 cytokines, whereas impaired Th1 immunity and the prevalent secretion of Th2 cytokines correlate with systemic and progressive disease (Kashino et al., 2000; Souto et al., 2000; Benard et al., 2001; Arruda et al., 2004, 2007; Pina et al., 2004). In PCM, the importance of Th17 immunity is not well defined. However, IL-17-expressing cells have been observed in cutaneous and mucosal lesions of PCM patients and have been associated with the organization of granulomas (Pagliari et al., 2011). It was also recently reported that the diverse patterns of T cell responses of *P. brasiliensis*-infected individuals lead to different clinical manifestations. The resistance to infection observed in asymptomatic individuals was shown to be mediated by a predominant Th1 response, which was responsible for macrophage activation. The most severe form of the disease, the juvenile form, presents a prevalent Th2/Th9 response, which is associated with an enhanced antibody response. The chronic inflammatory response characteristic of the adult form of the disease was associated with Th17 immunity, with important participation of Th1 cells (De Castro et al., 2013). In the murine model, our studies have previously shown that toll-like receptor (TLR)-2, TLR-4, and Dectin-1 regulate the development of Th17 and Tc17 cells against *P. brasiliensis* infection (Loures et al., 2009, 2010, 2014).

The contact of pathogens with host innate immune cells involves the interaction among multiple pathogen-associated molecular patterns (PAMPs) and their cognate pattern recognition receptors (PRRs), and it is this complex interaction that shapes the innate and adaptive responses of hosts (Trinchieri and Sher, 2007). In addition to β-1-3-glucans, the fungal cell wall is composed of many other components recognized by various PRRs that can exert synergistic or antagonistic effects on cell activation (Gantner et al., 2003; Netea et al., 2006). Gantner et al. provided the first demonstration that the cooperative signaling between TLR and non-TLR receptors (Dectin-1 and TLR-2) was required for TNF-α production by zymosan-stimulated macrophages (Gantner et al., 2003). Although Dectin-1 has a special ability to induce cytokine production by macrophages and DCs, it may also interact with other MyD88-coupled TLRs (TLR-2, TLR-4, TLR-5, TLR-7, TLR-9), resulting in the synergistic induction of several cytokines, including TNF-α, IL-10, IL-6, and IL-23 (Dennehy et al., 2008; Ferwerda et al., 2008). Interestingly, these interactions also control the down-regulation of IL-12 (Gerosa et al., 2008) and the reciprocal regulation of IL-23 and IL-12 that are essential for the development of Th17 responses mediated by Dectin-1 (LeibundGut-Landmann et al., 2007). Here, we report that curdlan, a specific Dectin-1 agonist, enhances lymphocyte activation, proliferation, and development of IL-17-producing T cells induced by *P. brasiliensis*-stimulated DCs. Accordingly, the absence of Dectin-1 expression induces decreased lymphocyte activation and proliferation in addition to impaired Th17 and Tc17 immune responses. Furthermore, studies with PRR-deficient cells allowed us to demonstrate that Dectin-1, mannose receptor (MR), TLR-2, and TLR-4 control lymphocyte proliferation and IL-17 production induced by *P. brasiliensis*-stimulated DCs. However, a synergist action on Th17 and Tc17 development was only mediated by Dectin-1, TLR-4 and MR.

## Materials and Methods

### Fungus

*Paracoccidioides brasiliensis* (*P. brasiliensis* 18 strain), a highly virulent isolate, was used throughout this investigation (Kashino et al., 1985). *P. brasiliensis* yeast cells were maintained by weekly sub-cultivation in semisolid culture medium at 37°C. Washed yeast cells were adjusted to 4 × 10^4^ cells/mL based on hemocytometer counts. Viability was determined with Janus Green B vital dye (Merck, Darmstadt, Germany) and was always higher than 85%.

### Mice

Eight- to 12-week-old male C57BL/6 (backcrossed for at least nine generations) *Clec7a*^–/–^ (Dectin-1 ^–/–^), TLR-2^–/–^, TLR-4^–/–^, and WT mice were obtained from the specific pathogen free Isogenic Breeding Unit of the Department of Immunology, Institute of Biomedical Sciences, University of São Paulo. Dectin-1^–/–^ mice were generously provided by Dr. Gordon Brown from the University of Aberdeen, UK. All animal procedures were approved by the Ethics Committee on Animal Experiments of the Institute of Biomedical Sciences of University of São Paulo (Proc.76/04/CEEA).

### DC Generation and Maturation *in vitro*

Bone-marrow derived DCs (BMDCs) were generated according to previously described methods (Inaba et al., 1992) with some modifications. Briefly, cells removed from femurs of non-infected mice were cultured with 20 ng/mL recombinant granulocyte–macrophage colony-stimulating factor (rGM-CSF; BD Bioscience; San Jose, CA, USA) and 2 ng/mL interleukin-4 (rIL-4; BD Bioscience) in complete medium RPMI (Difco, Detroit, MI, USA) containing 10% fetal calf serum, 2 mM L-glutamine, 100 U/mL penicillin, and 100 μg/mL streptomycin (Sigma, Germany). The medium containing rGM-CSF and rIL-4 was renewed on days 2 and 4 of culture. On day 6, highly purified DCs (CD11c^+^ cells) were obtained from culture by two rounds of positive selection using anti-CD11c-coated magnetic beads (Miltenyi Biotec, Cologne, Germany) and matured by 2 h incubation with *P. brasiliensis* yeasts at a DC: *P. brasiliensis* ratio of 10:1. Then, DCs were co-cultured with splenic lymphocytes from uninfected mice. The DC:lymphocyte ratio used was 1:10. Lymphocyte activation, cell division index (CDI) and the presence of intracellular cytokines were determined after 5 days of DC-lymphocyte co-culture.

### DC Treatment

Bone-marrow derived DCs from WT mice obtained as described above were treated with curdlan (100 μg/mL, Sigma, Germany) for 30 min prior to *P. brasiliensis* infection. After treatment, the DCs were infected with *P. brasiliensis* at a DC: *P. brasiliensis* ratio of 10:1 for 2 h and were then used in co-culture with naïve splenic lymphocytes. After 5 days, the proliferation, activation and Th17 differentiation of lymphocytes were evaluated. In another set of experiments, DCs from Dectin-1^–/–^, TLR-2^–/–^, TLR-4^–/–^, and WT mice were treated with anti-Dectin-1 (100 μg/mL; Serotec; Raleigh, NC, USA) or anti-MR (200 μg/mL; Serotec) antibodies for 30 min prior to *P. brasiliensis* infection. Following antibody treatment, the DCs were infected with *P. brasiliensis* and co-cultured with naïve splenic lymphocytes as described above. The CDI and differentiation were evaluated after 5 days of co-cultivation.

### Proliferation Assays

Lymphocytes were assayed for proliferation using an *in vitro* fluorescence-based assay. Briefly, 1 × 10^6^ cells from spleens of uninfected Dectin-1^–/–^, TLR-2^–/–^, TLR-4^–/–^, and WT mice were stained with 1 μL (5 mM) carboxyfluorescein diacetate succinimidyl ester (CFSE; Molecular Probes; Waltham, MA, USA) in PBS and 5% fetal calf serum for 15 min at room temperature. CFSE-stained cells were cultured for 5 days with *P. brasiliensis*-infected DCs, which were previously treated or untreated with curdlan or antibodies as described above. Lymphocytes were then stained with anti-CD4 and anti-CD8 antibodies (eBiosciences; San Diego, CA, USA). A minimum of 100,000 events was acquired on a FACSCANTO flow cytometer using FACSDIVA software (BD Biosciences; San Jose, CA, USA). The analyses were performed using FlowJo software (Tree Star; Ashland, OR, USA). The CDI was calculated as previously described by Mannering et al. (2003) and was based on the number of CFSE^+^CD4^+^ or CFSE^+^CD8^+^ T cells found in the stimulated culture/number of CFSE^+^CD4^+^ or CFSE^+^CD8^+^ T cells in the unstimulated culture.

### Cell Surface Markers and Intracellular Staining

Bone-marrow derived DCs from WT mice were treated with curdlan (100 μg/mL) for 30 min prior to *P. brasiliensis* infection. After treatment, the DCs were infected with *P. brasiliensis* at a DC: *P. brasiliensis* ratio of 10:1 overnight. The cells were harvested and stained by anti-Dectin-1, TLR-2, TLR-4, and MR antibodies (eBioscience and R&D Systems; Minneapolis, MN, USA). For intracellular cytokine evaluation, DC-lymphocyte co-cultures from Dectin-1^–/–^, TLR-2^–/–^, TLR-4^–/–^, and WT mice were stimulated for 6 h in complete medium in the presence of 50 ng/mL phorbol 12-myristate 13-acetate, 500 ng/mL ionomycin (both from Sigma-Aldrich; Germany) and monensin (3 mM, eBioscience). After surface staining for CD4, CD8, CD25, and CD69, the cells were fixed, permeabilized, and stained with anti-IL-17 antibodies (eBioscience). The cell surface expression of leukocyte markers and the presence of intracellular IL-17 were assessed by flow cytometry using a FACSCANTO flow cytometer and the FACSDIVA software (BD Pharmingen). A minimum of 100,000 events was acquired. The analyses were performed using FlowJo software (Tree Star).

### Measurement of Cytokines

Bone-marrow derived DCs from WT mice were treated or not with curdlan (100 μg/mL) for 30 min prior to *P. brasiliensis* infection. After treatment, the DCs were infected or not with *P. brasiliensis* at a DC: *P. brasiliensis* ratio of 10:1 for 2 h and were used in co-culture with naïve splenic lymphocytes. After 5 days of co-culture, the supernatants were removed and stored at -70°C. The levels of cytokines (IL-23, IL-17, IL-6 and TNF-α) were measured by capture enzyme-linked immunosorbent assay (ELISA) with antibody pairs purchased from eBioscience. The ELISA procedure was performed according to the manufacturer’s protocol, and absorbance was measured with a Versa Max Microplate Reader (Molecular Devices; Sunnyvale, CA, USA).

### Quantitative Real-Time PCR

Total RNA was extracted from DC-lymphocyte co-cultures using TRIzol reagent (Invitrogen, Life Technologies; Waltham, MA, USA) according to the manufacturer’s instructions. RNA concentrations were determined by spectrophotometer (Nanodrop; Thermo Scientific; Waltham, MA, USA) readings at an absorbance of 260 nm. First-strand cDNA was synthesized from 2 μg RNA using the High Capacity RNA-to-cDNA kit (Applied Biosystems; Waltham, MA, USA) according to the manufacturer’s instructions. Real-time polymerase chain reaction (RT-PCR) was performed using the TaqMan real-time PCR assay (Applied Biosystems) for the RAR-related orphan receptor γ (RORγt; Mm012661022_m1; Applied Biosystems) transcription factor. Cycling conditions were as follows: 10 min at 95°C, followed by 40 cycles of 30 s at 95°C, 60 s at 55°C, and 60 s at 72°C. Analysis was performed with the Stratagene MX3500p sequence detection system (Agilent Technologies; Santa Clara, CA, USA). Glyceraldehyde-3-phosphate dehydrogenase (GAPDH) (Mm03302249_g1; Applied Biosystems) was used as an internal control. All values were normalized to GAPDH, and the relative gene expression was calculated using the Pfaffl method (Pfaffl, 2001).

#### Statistical Analysis

For comparisons of two groups, means ± standard deviations (SD) were analyzed using a two-tailed unpaired Student’s *t*-test with the Tukey correction test. For comparisons of greater than two groups, significance was determined using the one- or two-way analysis of variance (ANOVA) with Tukey correction test. Calculations were performed using statistical software (GraphPad Prism 5.01). *P* values ≤ 0.05 were considered significant.

## Results

### Curdlan, a Specific Dectin-1 Agonist, Induces Enhanced Lymphocyte Proliferation, Activation, and Development of IL-17-Producing T cells

**FIGURE 1:**
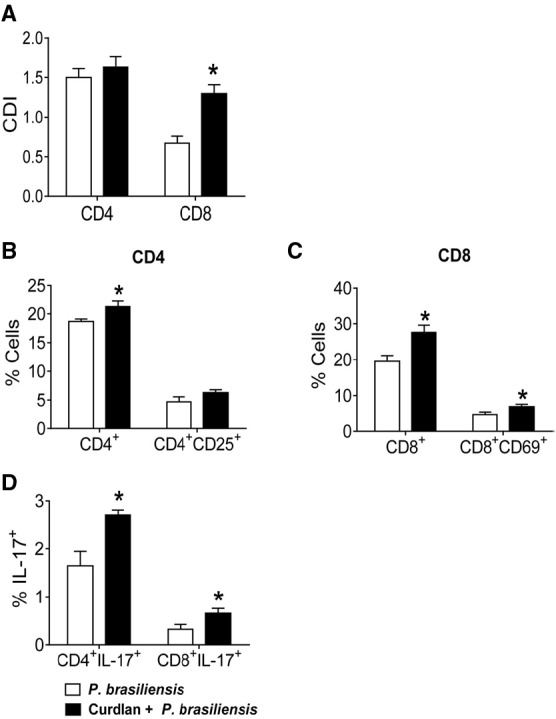
**Influence of curdlan, a Dectin-1 agonist, in T cell proliferation, activation and development of IL-17 producing T cells.** BMDCs of WT C57BL/6 mice were cultured in the presence of GM-CSF and IL-4 for 5 days. The DCs were then treated or not with curdlan (100 μg/ml) for 30 min and infected with viable *P. brasiliensis* yeasts for 2 h. Splenic lymphocytes from uninfected C57BL/6 mice were labeled with CFSE (5 mM) and co-cultivated with curdlan treated and untreated infected DCs. After 5 days, the cells were adjusted to 1 × 10^6^, labeled with specific anti-CD4, CD8, CD25, and CD69 antibodies and analyzed by flow cytometry. **(A)** Proliferative response of CD4^+^ and CD8^+^ T cells. **(B,C)** The lymphocyte population was gated by FSC/SSC analysis and gated cells were analyzed for CD4^+^CD25^+^ **(B)** and CD8^+^CD69^+^ **(C)** expression. Some cells were re-stimulated with PMA/ionomycin for 6 h and subjected to intracellular staining for IL-17 **(D)**. The results are expressed as frequency of positive cells. Data are means ± SD of five wells per group from thee independent experiments (*P < 0.05).

To determine the involvement of Dectin-1 receptor in the modulation of the immune response against *P. brasiliensis*, BMDCs from C57BL/6 mice were treated or not with curdlan for 30 min and infected with viable *P. brasiliensis* yeast cells for 2 h. In parallel, splenic lymphocytes from uninfected C57BL/6 mice were previously labeled with CFSE and co-cultured with infected DCs. After 5 days, proliferation and the phenotypes of lymphocytes were analyzed by flow cytometry. An increased proliferative response of CD8^+^ but not CD4^+^ T cells was observed in curdlan-treated cultures (Figure 1A). In addition, increased frequencies of CD4^+^, CD8^+^, and CD8^+^CD69^+^ T cells were observed when DC-lymphocyte cultures were pre-treated with curdlan (Figures 1B,C). Curdlan treatment also induced a higher frequency of CD4^+^IL-17^+^ (Th17) and CD8^+^IL-17^+^ (Tc17) cells when compared with curdlan untreated cultures (Figure 1D).

### Curdlan Enhances the Synthesis of IL-17, IL-6, and TNF-α Concomitantly with Increased Expression of RORγt

**FIGURE 2:**
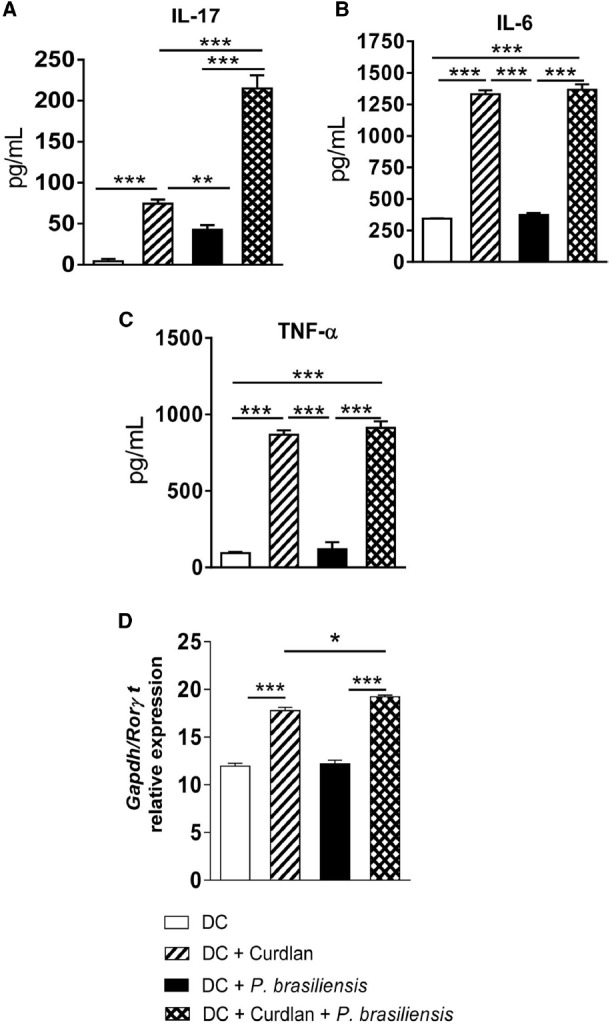
**Curdlan enhances the synthesis of IL-17, IL-6, and TNF-α concomitantly with increased mRNA expression of RORγt.** BMDCs of WT C57BL/6 mice were cultured as described in materials and methods. The cells were then treated or not with curdlan (100 μg/ml) for 30 min and infected with viable *P. brasiliensis* yeasts for 2 h. Splenic lymphocytes from uninfected C57BL/6 mice were co-cultivated with curdlan treated and untreated infected DCs. After 5 days, the supernatants were removed and used for cytokine measurements by ELISA **(A–C)**. Total RNA was extracted from cell cultures and RORγt mRNA measured using TaqMan real-time PCR assay. All values were normalized to Glyceraldehyde-3-phosphate dehydrogenase (GAPDH) that was used as an internal control. **(D)** Data are means ± SD of five wells per group from two independent experiments (*P < 0.05; **P < 0.01; ***P < 0.001).

The supernatants of the lymphocyte-DC co-cultures showed higher levels of IL-17, IL-6, and TNF-α following curdlan treatment (Figures 2A–C). mRNA expression of the transcription factor RORγt was also analyzed and was in accordance with our cytokine data (Figure 2D). This result demonstrates that additional stimulation of *P. brasiliensis*-activated DCs by a Dectin-1 agonist results in enhanced expression of RORγt mRNA, the transcription factor involved in the differentiation of IL-17-producing T cells.

### Curdlan Regulates the Expression of Innate Immune Receptors in *P. brasiliensis*-stimulated DCs

**FIGURE 3:**
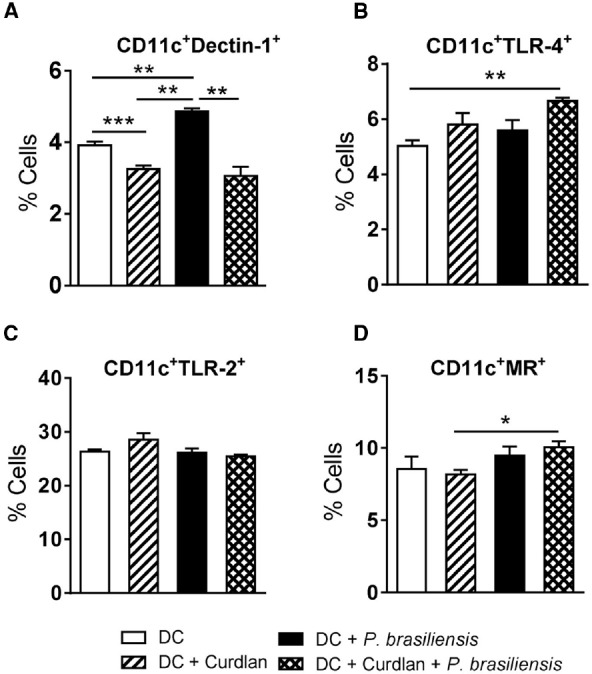
**Curdlan regulates the expression of innate immune receptors in *P. brasiliensis*-stimulated DCs.** BMDCs from WT C57BL/6 mice were cultured as previously described. The cells were then treated or not with curdlan (100 μg/ml) for 30 min and infected with viable *P. brasiliensis* yeasts overnight. The cells were adjusted to 1 × 10^6^, labeled with specific anti-CD11c, Dectin-1, TLR-2, TLR-2, and MR antibodies and analyzed by flow cytometry. The leukocyte population was gated by FSC/SSC analysis and CD11c^+^ gated cells were analyzed for CD11c^+^Dectin-1^+^ **(A)**, CD11c^+^TLR-4^+^ **(B)**, CD11c^+^TLR-2^+^ **(C)**, and CD11c^+^MR^+^ expression **(D)**. The results are expressed as frequency of positive cells. Data are means ± SD of five wells per group from two independent experiments (*P < 0.05; **P < 0.01; ***P < 0.001).

To determine the involvement of curdlan in the modulation of innate immune receptors in DCs, BMDCs from C57BL/6 mice were treated or not with curdlan for 30 min and infected with viable *P. brasiliensis* yeast cells overnight. The expression of Dectin-1, TLR-2, TLR-4, and MR was then assessed by flow cytometry. Curdlan treatment caused a decreased frequency of Dectin-1 by *P. brasiliensis*-infected or uninfected DC cultures (Figure 3A). However, an increased frequency of DCs expressing TLR-4 and MR was detected only when the DCs were concomitantly treated with curdlan and *P. brasiliensis* yeasts (Figures 3B,D). The expression of TLR-2 was not modulated by curdlan in infected or uninfected DCs (Figure 3C).

### Expression of Dectin-1 Induces Increased Lymphocyte Proliferation, Activation, and Development of IL-17-Producing T cells

**FIGURE 4:**
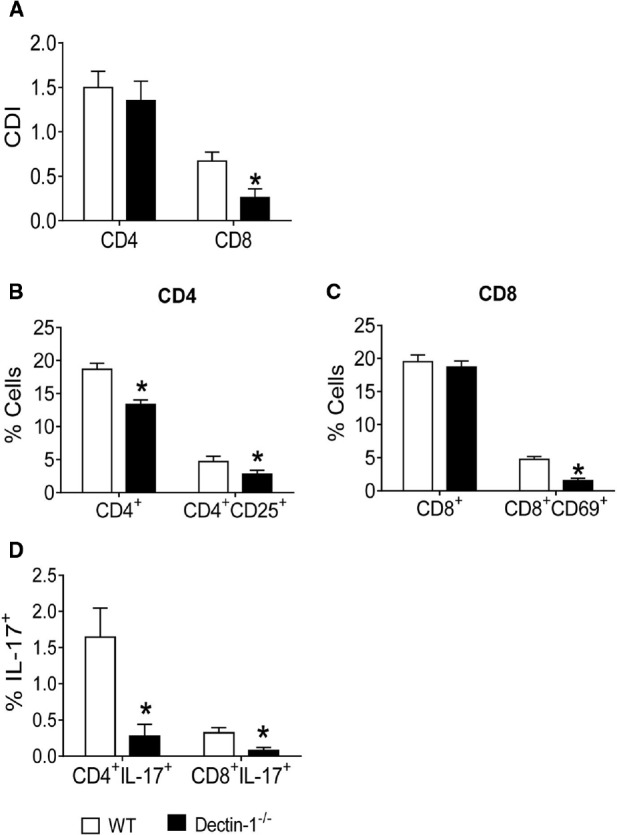
**Influence of Dectin-1 receptor in the lymphocyte proliferation, activation and development of IL-17 producing T cells.** BMDCs from WT and Dectin-1^–/–^ C57BL/6 mice were cultured as described and then infected with viable *P. brasiliensis* yeasts for 2 h. Splenic lymphocytes from uninfected WT and Dectin-1^–/–^ mice were previously labeled with CFSE (5 mM) and co-cultivated with DCs. After 5 days, the cells were adjusted to 1 × 10^6^, labeled with specific anti-CD4, CD8, CD25, and CD69 antibodies and analyzed by flow cytometry. **(A)** Proliferative response of CD4^+^ and CD8^+^ T cells. **(B,C)** The leukocyte population was gated by FSC/SSC analysis and gated cells were analyzed for CD4^+^CD25^+^ **(B)** and CD8^+^CD69^+^ **(C)** expression. Some cultures were re-stimulated with PMA/ionomycin for 6 h and subjected to intracellular staining for IL-17. The results are expressed as frequency of IL-17-positive cells **(D)**. Data are means ± SD of five wells per group from three independent experiments (*P < 0.05).

To better evaluate the role of Dectin-1 in the activation of lymphocytes and differentiation of Th17/Tc17 cells, Dectin-1^–/–^ and WT mice were used in co-culture experiments as described above. Thus, BMDCs from WT and Dectin-1^–/–^ mice were infected with viable *P. brasiliensis* yeast cells for 2 h. In parallel, splenic lymphocytes from uninfected WT and Dectin-1^–/–^ mice were previously labeled or not with CFSE and co-cultured with *P. brasiliensis*-infected DCs. After 5 days, lymphocytes were analyzed by flow cytometry. Lower proliferative responses of CD8^+^ T cells were observed in Dectin-1^–/–^ cultures in comparison with Dectin-1-sufficient WT cultures. No differences in the proliferation of CD4^+^ T cells were observed between the two analyzed groups (Figure 4A). Using Dectin-1^–/–^ cells, a lower frequency of CD4^+^, CD4^+^CD25^+^, and CD8^+^CD69^+^ T lymphocytes (Figures 4B,C) was observed when compared with WT cells. Furthermore, a lower frequency of IL-17-producing CD4^+^ and CD8^+^ T cells was expanded in Dectin-1^–/–^ cultures (Figure 4D). Combined, our data indicate that Dectin-1 is important for the proliferation of T cells and the induction of Th17/Tc17 cells by *P. brasiliensis*-infected DCs. Furthermore, even in the absence of Dectin-1 expression, some Th17/Tc17 cells were expanded, indicating that other PRRs may be involved in this response. This led us to investigate the cooperation among TLR-2, TLR-4, Dectin-1, and MR in the activation of T cells and differentiation of IL-17-producing cells.

### Dectin-1 Collaborates with TLR-4 in the Induction of T cell Proliferation, Activation, and Development of IL-17-Producing cells

**FIGURE 5:**
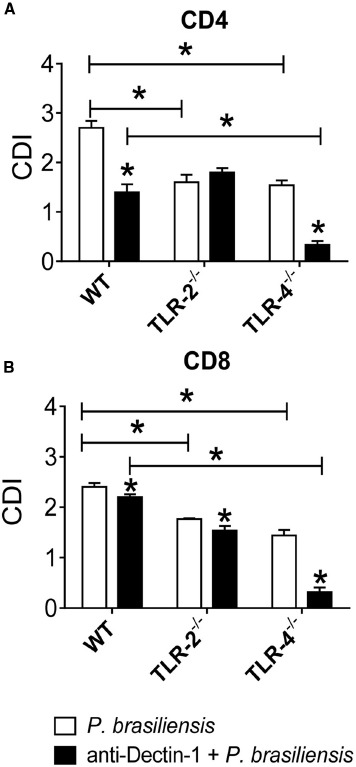
**Dectin-1 collaborates with TLR-4 in the induction of T cell proliferation.** BMDCs from WT, TLR-2^–/–^, and TLR-4^–/–^ mice were treated or not with anti-Dectin-1 antibody for 30 min and infected with viable *P. brasiliensis* yeasts for 2 h. Splenic lymphocytes from uninfected WT, TLR-2^–/–^, and TLR-4^–/–^ mice were previously labeled with CFSE (5 mM) and co-cultured with anti-Dectin-1 treated or untreated DCs. After 5 days, the cells were adjusted to 1 × 10^6^, labeled with specific anti-CD4 **(A)** and CD8 **(B)** antibodies and analyzed by flow cytometry. The lymphocyte population was gated by FSC/SSC analysis. The results are expressed as frequency of positive cells. Data are means ± SD of five wells per group from three independent experiments (*P < 0.05).

To assess the cooperation of innate receptors in the proliferation and differentiation of lymphocytes induced by *P. brasiliensis*-activated DCs, BMDCs from WT, TLR-2^–/–^, and TLR-4^–/–^ mice were treated or not with anti-Dectin-1 antibodies and co-cultured with naïve splenic lymphocytes as described previously. In the absence of TLR-2 or TLR-4, the proliferation of CD4^+^ T cells was impaired. Moreover, a significant difference was found between anti-Dectin-1-treated WT and TLR-4^–/–^ cultures, indicating that TLR-4 and Dectin-1 cooperate in the induction of lymphoproliferative responses (Figure 5A). The data obtained for TLR-2^–/–^ and TLR-4^–/–^ cells demonstrated that these PRRs participate in the proliferative responses of CD8^+^ T cells, and the use of anti-Dectin-1 antibodies revealed the co-participation of TLR-4 and Dectin-1 in the proliferative responses of CD8^+^ T lymphocytes induced by *P. brasiliensis-*matured DCs (Figure 5B).

**FIGURE 6:**
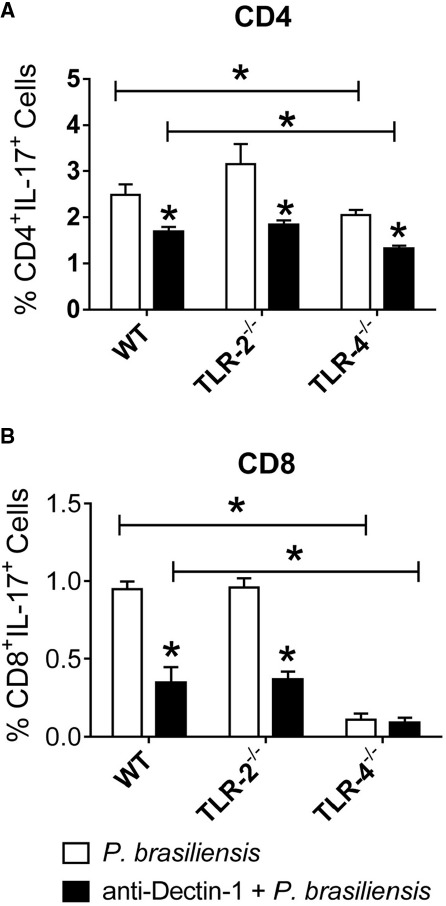
**Dectin-1 collaborates with TLR-4 in the development of IL-17 producing cells.** BMDCs from WT, TLR-2^–/–^, and TLR-4^–/–^ mice were treated or not with anti-Dectin-1 antibody for 30 min and infected with viable *P. brasiliensis* yeasts for 2 h. Splenic lymphocytes from uninfected WT, TLR-2^–/–^, and TLR-4^–/–^ mice were co-cultivated with anti-Dectin-1 treated or untreated DCs. After 5 days, the cells were adjusted to 1 × 10^6^ and re-stimulated with PMA/ionomycin for 6 h, labeled with specific anti-CD4 **(A)** and CD8 **(B)** antibodies and subjected to intracellular staining for IL-17. The cells were analyzed by flow cytometry, and the lymphocyte population gated by FSC/SSC analysis. The results are expressed as frequency of positive cells. Data are means ± SD of five wells per group from three independent experiments (*P < 0.05).

Next, we examined the cooperation of innate immune receptors in the induction of Th17 and Tc17 lymphocytes. As shown in Figure 6A, the absence of TLR-4 expression and Dectin-1 blockade by specific antibodies reduced the differentiation of Th17 cells. In addition, a decreased frequency of Th17 cells was observed in anti-Dectin-1-treated WT, TLR-2^–/–^, and TLR-4^–/–^ co-cultures, but a synergistic effect was only observed between Dectin-1 and TLR-4 receptors. Indeed, anti-Dectin-1-treated TLR-4^–/–^ cultures expanded a significantly lower frequency of Th17 lymphocytes than anti-Dectin-1-treated WT cultures. Regarding the differentiation of Tc17 cells, a marked influence of TLR-4 and Dectin-1 was noted. Dectin-1 blockade impaired Tc17 differentiation by WT and TLR-2^–/–^ cells, but it did not influence the already low differentiation observed in TLR-4^–/–^ co-cultures (Figure 6B). The significant difference in the frequencies of Tc17 lymphocytes between anti-Dectin-1-treated WT and TLR-4^–/–^ cells indicates the cooperation between Dectin-1 and TLR-4. Together, these findings indicate the synergistic contribution of Dectin-1 and TLR-4 in the differentiation of both Th17 and Tc17 cells.

### MR Cooperates with TLR-4 in the Proliferation of CD4^+^ and CD8^+^ T lymphocytes and in the Differentiation of TH17/TC17 cells

**FIGURE 7:**
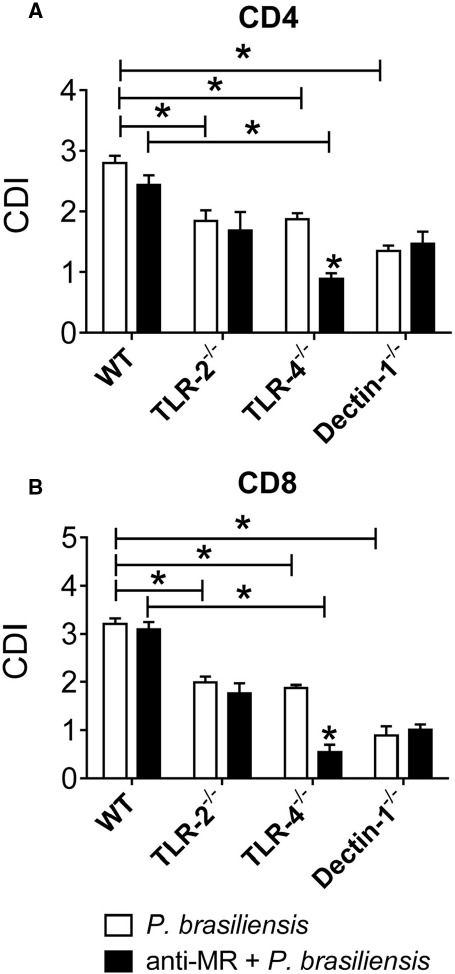
**MR collaborates with TLR-4 in the induction of T cell proliferation.** BMDCs from WT, TLR-2^–/–^, TLR-4^–/–^, and Dectin-1^–/–^ mice were cultured and then treated or not with anti-MR antibody for 30 min and infected with viable *P. brasiliensis* yeasts for 2 h. Splenic lymphocytes from uninfected WT, TLR-2^–/–^, TLR-4^–/–^, and Dectin-1^–/–^ mice were previously labeled with CFSE (5 mM) and co-cultivated with anti-MR treated or untreated DCs. After 5 days, the cells were adjusted to 1 × 10^6^, labeled with specific anti-CD4 **(A)** and CD8 **(B)** antibodies and analyzed by flow cytometry. The lymphocyte population was gated by FSC/SSC analysis. The results are expressed as frequency of positive cells. Data are means ± SD of five wells per group from three independent experiments (*P < 0.05).

We next examined the cooperation of MRs with TLR-2, TLR-4, and Dectin-1 in the proliferative response of T cells and in the differentiation of naïve T cells to the Th17/Tc17 phenotypes by *P. brasiliensis*-challenged DCs. The same experimental protocol was employed except an anti-MR antibody was used to treat cultures. In untreated cultures, the collaboration of TLR-2, TLR-4, and Dectin-1 was once more observed in the proliferation of CD4^+^ and CD8^+^ T lymphocytes (Figures 7A,B). Anti-MR treatment reduced the proliferation of CD4^+^ and CD8^+^ T cells from TLR-4^–/–^ mice (Figures 7A,B). These data indicate that TLR-4 cooperates with MRs in the activation of proliferative responses of CD4^+^ and CD8^+^ T cells induced by *P. brasiliensis*-stimulated DCs.

**FIGURE 8:**
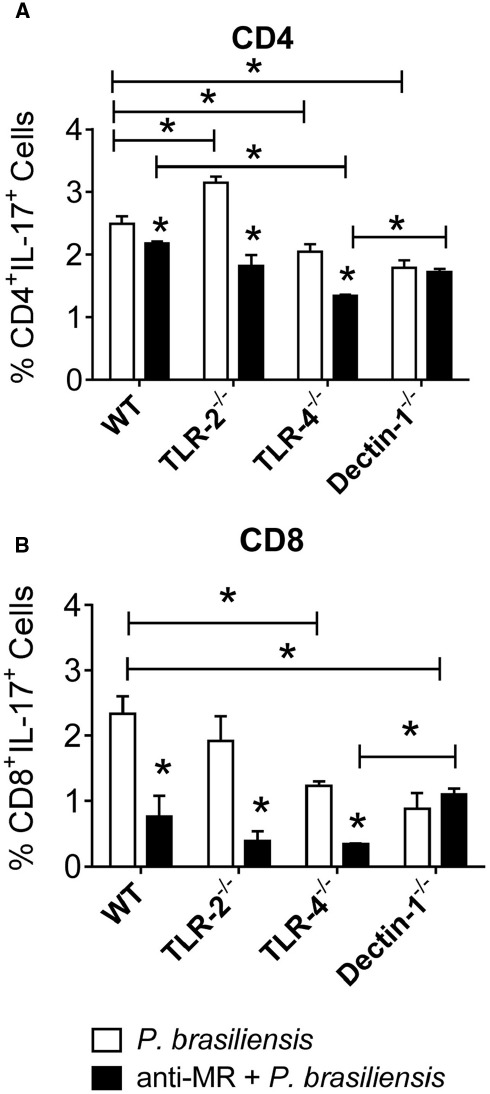
**MR collaborates with TLR-4 in the development of IL-17 producing cells.** BMDCs from WT, TLR-2^–/–^, TLR-4^–/–^, and Dectin-1^–/–^ mice were cultured and then treated or not with anti-MR antibody for 30 min, and infected with viable *P. brasiliensis* yeasts for 2 h. Splenic lymphocytes from uninfected WT, TLR-2^–/–^, TLR-4^–/–^, and Dectin-1^–/–^ mice were co-cultures with anti-MR treated or untreated DCs. After 5 days, the cells were adjusted to 1 × 10^6^ and re-stimulated with PMA/ionomycin for 6 h, labeled with specific anti-CD4 **(A)** and CD8 **(B)** antibodies and subjected to intracellular staining for IL-17. The cells were analyzed by flow cytometry, and lymphocyte population gated by FSC/SSC analysis. The results are expressed as frequency of positive cells. Data are means ± SD of five wells per group from three independent experiments (*P < 0.05).

With untreated cultures, an inhibitory function of TLR-2 was detected in the differentiation of Th17 cells. The use of anti-MR antibodies allowed us to show the synergistic action of TLR-4 and MR in Th17 expansion (Figure 8A). In addition, cooperation between TLR-4 and MR was also observed in Tc17 differentiation (Figure 8B).

## Discussion

Toll-like receptor and CLR signaling pathways are essential for immunity against fungal pathogens. The immune responses triggered by these receptors include phagocytosis, induction of antifungal effector mechanisms and the production of various soluble mediators, including cytokines, chemokines and lipid mediators (Calich et al., 2008; Robinson et al., 2009; Hardison and Brown, 2012; Loures et al., 2012; Kawasaki and Kawai, 2014). These receptors also have an important role in the development of adaptive immunity, particularly Th1 and Th17 responses (Gringhuis et al., 2007; van de Veerdonk et al., 2009; Saijo et al., 2010). In pulmonary PCM, our group demonstrated that TLR-2 negatively regulates Th17 immunity. TLR-2^–/–^ mice develop a prevalent Th17 immunity that controls fungal growth but also induces tissue pathology mediated by neutrophils (Loures et al., 2009). In contrast, TLR-4-defective mice developed an inefficient immune response concomitant with decreased expansion of Th17 lymphocytes and elevated differentiation of Treg cells (Loures et al., 2010). Another PRR, Dectin-1, was also shown to play an important role in the control of Treg/Th17 immunity to *P. brasiliensis* infection. Dectin-1^–/–^ mice developed a more severe infection that resulted in progressive pathogen-mediated tissue pathology and increased mortality. Moreover, the absence of Dectin-1 signaling increased Treg cell expansion and inhibited the differentiation of IL-17^+^ CD8^+^ (Tc17) T cells (Loures et al., 2014).

Curdlan, a specific agonist of Dectin-1 (Goodridge et al., 2009; Horiuchi et al., 2012), was used here to assess the role of Dectin-1 in lymphocyte activation and Th17/Tc17 differentiation induced by *P. brasiliensis*-challenged DCs. We verified that *P. brasiliensis* by itself was able to induce T cell proliferation and activation as well as Th17/Tc17 differentiation, but the concomitant activation by curdlan enhanced these T cell responses. The increased secretion of IL-17 and the enhanced mRNA expression of the transcription factor RORγt in curdlan-stimulated infected DCs confirmed the role of Dectin-1 in the differentiation of Th17/Tc17 cells. This finding is in agreement with other studies demonstrating that curdlan-stimulated DCs are efficient at priming naïve CD4^+^ and CD8^+^ T cells to differentiate into Th17/Tc17 and Th1 cells (Iezzi et al., 2009; Agrawal et al., 2010; Higashi et al., 2010; Ghosh et al., 2013). Furthermore, curdlan was also shown to contribute to the expression of several innate immune receptors in *P. brasiliensis-*infected DCs. As demonstrated here, an increased frequency of DCs expressing TLR-4 and MR was detected in DC cultures treated with curdlan and infected with *P. brasiliensis* yeasts. This increased TLR-4 expression is in agreement with our previous report demonstrating that lung cells of Dectin-1^–/–^ mice present a lower frequency of F4/80^+^TLR-4^+^ cells than WT mice (Loures et al., 2014). Curdlan treatment also caused a decreased frequency of Dectin-1^+^ cells by *P. brasiliensis*-infected or uninfected DCs. The interaction between curdlan and Dectin-1 was previously shown to cause Dectin-1 internalization (Goodridge et al., 2009, 2011), which may explain the decreased frequency of Dectin-1^+^ DCs observed in this study. The importance of Dectin-1 was further determined using Dectin-1^–/–^ mice. Dectin-1 expression by DCs induced increased lymphocyte activation, proliferation, and development of IL-17-producing T cells. This is in agreement with our *in vivo* studies demonstrating that lung lymphocytes from *P. brasiliensis*-infected Dectin-1^–/–^ mice produced lower levels of IL-17 when compared with lymphocytes from WT mice (Loures et al., 2014).

To better evaluate the collaboration of diverse PRRs in the induction of T cell differentiation, naïve lymphocytes from WT, TLR-2^–/–^, TLR-4^–/–^, and Dectin-1^–/–^ C57BL/6 mice were cultured with *P. brasiliensis-*challenged DCs previously treated or untreated with anti-Dectin-1 antibody. Our results demonstrated that the absence of TLR-2, TLR-4, and Dectin-1 impaired the proliferation of T cells. These data are similar to those obtained by our group when lymphocytes from TLR-2- and TLR-4-sufficient and -deficient mice were stimulated with anti-CD3 and anti-CD28 antibodies (Loures et al., 2009, 2010). It is worth remembering that the stimulus for lymphoproliferation was *P. brasiliensis*-infected DCs previously treated or not with anti-Dectin-1 antibody. Reduced lymphocyte proliferation was observed in anti-Dectin-1 treated DCs, indicating the involvement of this receptor in lymphocyte proliferative activity. Furthermore, an additional reduction in lymphocyte proliferation was observed in anti-Dectin-1-treated TLR-4^–/–^ cells, indicating synergistic action between TLR-4 and Dectin-1.

In this study, we also demonstrated how TLR-2 and TLR-4 act in synergy with Dectin-1 to determine Th17 differentiation. The literature is controversial regarding the main receptors involved in the induction of Th17 immune responses. Some groups have reported the role of TLRs in this event (Evans et al., 2007; Loures et al., 2010), although others have highlighted the importance of Dectin-1 (LeibundGut-Landmann et al., 2007; Loures et al., 2014), NOD2 (van Beelen et al., 2007), or MR (van de Veerdonk et al., 2009). In general, these studies suggest that several PRRs (TLRs or not) may act independently or in synergy for Th17 differentiation (van de Veerdonk et al., 2009; Zenaro et al., 2009; Chang et al., 2014) and control of inflammatory responses against fungal microorganisms, including *Candida albicans* (van de Veerdonk et al., 2010; Le et al., 2012; Ifrim et al., 2013). Without antibody treatment, reduced frequencies of CD4^+^IL-17^+^ and CD8^+^IL-17^+^ lymphocytes were found in TLR-4^–/–^ and Dectin-1^–/–^ cells, indicating the involvement of these receptors in the induction of Th17 and Tc17 responses. Furthermore, anti-Dectin-1 treatment of DCs drastically reduced the production of IL-17, and this was even lower with TLR-4^–/–^ cells co-cultured with anti-Dectin-1-treated DCs. These data demonstrate the synergistic action of TLR-4 and Dectin-1 in the induction of Th17 immune responses. These findings are in agreement with our previous reports showing the involvement of these receptors in the induction of Th17 and Tc17 immune responses (Loures et al., 2010, 2014). We have demonstrated that lung lymphocytes from *P. brasiliensis*-infected TLR-4^–/–^ and Dectin-1^–/–^ mice produce lower levels of IL-17 compared with lymphocytes from WT mice. However, we demonstrated here for the first time the synergistic action of these receptors in the induction of Th17 cells.

Our results are also in agreement with recent publications describing the individual or synergistic action of Dectin-1 in the induction of Th17 immune responses. A recent study clearly demonstrated that the concomitant activation of Dectin-1 and the intracytoplasmic inflammasome is essential for Th17 immunity against C. *albicans* infection (Cheng et al., 2011). In addition, Dennehy et al. (2009) reported that the induction of Th17 immunity to *C. albicans* was the result of synergistic action between TLR-2 and Dectin-1. In a model of *Mycobacterium tuberculosis* infection, van de Veerdonk et al. (2009) reported that Dectin-1 and TLR-4 are involved in IL-17 production, and the blockade of other receptors, such as NOD2, TLR-2, and MR, had no effect on this activity.

Next we studied the synergistic effect of TLR-2, TLR-4, Dectin-1, and MR on the induction of IL-17-producing cells using DCs treated or untreated with anti-MR. Again, a lower frequency of CD4^+^IL17^+^ and CD8^+^IL17^+^ lymphocytes was expanded when TLR-4^–/–^ and Dectin-1^–/–^ cells were used. Moreover, treatment of DCs with anti-MR inhibited the production of IL-17 because a lower frequency of CD4^+^IL-17^+^ and CD8^+^IL-17^+^ cells was observed in all treated groups. This inhibition was greater with TLR-4^–/–^ cells, demonstrating the synergistic effect of TLR-4 not only with Dectin-1, but also with MR. Our data have also demonstrated the participation of MR in the induction of lymphocyte proliferation. This finding is consistent with other studies (van de Veerdonk et al., 2009) demonstrating that MRs of macrophages are involved in Th17 immunity against *C. albicans*. It was recently demonstrated that mannans and β-glucans of *C. albicans* are recognized by MR and Dectin-1/TLR-2, respectively, culminating in Th17-biased immune responses (Smeekens et al., 2010).

In murine PCM, the contribution of numerous PRRs appears to control innate and adaptive immunity. The results reported here indicate that Th17/Tc17 immunity generated by *P. brasiliensis*-infected hosts is mediated by the synergistic action of TLR-4, MR, and Dectin-1 expressed by DCs. These receptors may be activated by a number of fungal components present on fungal cell walls or actively secreted by infecting yeast cells.

### Conflict of Interest Statement

The authors declare that the research was conducted in the absence of any commercial or financial relationships that could be construed as a potential conflict of interest.
